# Lipid domains in HIV-1 assembly

**DOI:** 10.3389/fmicb.2014.00220

**Published:** 2014-05-19

**Authors:** Naresh Yandrapalli, Delphine Muriaux, Cyril Favard

**Affiliations:** Centre d'étude des Pathogènes et de Biotechnologies pour la Santé, CNRS UMR-5236Montpellier Cedex, France

**Keywords:** HIV-1, Gag, PIP2, lipid domains, lipid molecular shape

## Abstract

In CD^+^_4_ T cells, HIV-1 buds from the host cell plasma membrane. The viral Gag polyprotein is mainly responsible for this process. However, the intimate interaction of Gag and lipids at the plasma membrane as well as its consequences, in terms of lipids lateral organization and virus assembly, is still under debate. In this review we propose to revisit the role of plasma membrane lipids in HIV-1 Gag targeting and assembly, at the light of lipid membranes biophysics and literature dealing with Gag-lipid interactions.

## Introduction

In a very oversimplified view, assembling could be seen as: retrieving all of your partners at the right place on the right time. In the case of enveloped retroviruses such as HIV, this means retrieving in a sophisticated spatio-temporal concerted mechanism:

- genomic RNA and cellular t-RNA nucleotide primers
- structural (Gag and GagPol) and other (protease, transcriptase and integrase) proteins or polyproteins
- lipid membrane embedded envelop glycoproteins (Env).

in order to correctly produce a potential infective new virion.

The main molecular constituents of HIV-1 are Gag polyprotein (50% of the virion mass) and the viral envelop membrane lipids (30%) exclusively issued from the host cell (for review see Carlson et al., 2008). Gag is a polyprotein that has the ability to induce on its own the formation of virus like particle (VLPs) without any requirement of other viral or cellular components (except lipid membrane) (Gheysen et al., 1989; Campbell and Rein, 1999). Although Gag has been reported to interact with a cellular motor protein (Tang et al., 1999; Martinez et al., 2008) and with other components of vesicular trafficking pathway (Dong et al., 2005; Camus et al., 2007), it is not clear whether Gag is targeted to the plasma membrane or simply reaches the plasma membrane by diffusion through the cytosol. It has been shown that Gag molecules do not multimerize extensively before they reach the membrane (Kutluay and Bieniasz, 2010) and that they arrive at the plasma membrane as dimers or monomers that will multimerize onto eventual nucleation sites composed of Gag-RNA complexes (Jouvenet et al., 2009; Ku et al., 2013). Other major components of the viral infectivity are the Env glycoproteins, they reach the plasma membrane independently of Gag. Env is constituted of two different subunits gp120 and gp41, the later being a transmembrane protein. The gp41 protein is twice palmitoylated and is considered to be targeted to the so called lipid “rafts” membrane domains (Patil et al., 2010).

Undoubtedly, Gag is the main pillar of HIV assembly. It recruits the constituents of HIV virions and orchestrates their assembly while multimerizing onto the inner leaflet of the plasma membrane. Although assembly should appear as a very simple mechanism, many questions concerning Env recruitment and incorporation into virions remain unsolved. Different studies have shown that the cytoplasmic tail of the gp41 and the N-terminal part of Gag are both necessary for Env incorporation into virions, suggesting therefore an interaction of these two proteins, whether direct or through a cellular protein intermediate (for review see Murakami, 2008). Nevertheless, since HIV-1 only displays a tenth of glycoprotein trimers (Zhu et al., 2006) for thousands of Gag molecules (Turner and Summers, 1999; Briggs et al., 2004), it can still be questioned whether or not Gag and Env interact directly? Moreover, it is still to be known if there is a sequential order importance for the control of viral assembly.

In every polymerization by addition, the triggering of the reaction, called initiation step, is the formation of an activated monomer. The propagation of the polymerization will depend on local concentration of the “reactants” i.e., the number of monomers surrounding the activated monomer. Although it is not established that Gag multimerization needs an activated form of it, a simple guess shows that the reduction of dimensionality principle (Adam and Delbrück, 1968) should favor enhanced kinetics of the multimerization process. Therefore, it is clear that lipid membranes could play an important role in the assembly process. Moreover, the lateral compartmentalization of lipid membranes could locally induce higher concentrations of Gag monomers and facilitate the retrieval of Env proteins. Due to their physical and chemical heterogeneity, the detailed role of cellular plasma membrane in the assembly process of HIV-1 is still a source of controversy.

It is the aim of this review to remind basic concepts in lipid membrane organization and domains formation and to introduce the concept of lipid molecular shape and its consequence on the bilayer curvature. Thereafter, the role of lipids in HIV-1 assembly will be considered, by looking at the interaction of Gag with the plasma membrane at the molecular (atomistic) level. Finally the current model of HIV-1 assembly at the cell plasma membrane will be discussed.

## Plasma membrane: basic physical properties

### Lateral segregation and lipid domains

Our current view of plasma membrane mainly derives from the fluid mosaic model proposed 40 years ago by Singer and Nicolson (1972) arguing for proteins embedded in a homogeneous sea of lipids. Nevertheless the possibility of lipids segregating laterally to form “domains” in model membrane was reported at the same time (Phillips et al., 1970; Shimshick and McConnell, 1973). Later on, other types of membrane domains induced by protein-lipid interactions were proposed to explain membrane-mediated processes (Marcelja, 1976; Sackmann et al., 1984). Meanwhile, Israelachvili also proposed a model that accounts for the need of membrane proteins (peripheral and transmembrane) and lipids to adjust to each other due to packing effects as well as thermodynamics (i.e., adjust hydrophobic and hydrophilic areas and height) (Israelachvili, 1977), inducing thereby lateral heterogeneities. Based on this, Mouritsen and Bloom (1984) proposed the hydrophobic mismatch model where hydrophobic matching conditions can lead to elastic distortions of the lipid matrix, therefore, resulting in clustering of adapted lipid molecules around a transmembrane protein. Finally, another model accounting for the role of structural peripheral proteins and sugars (cytoskeleton and glycogalix) has been proposed by Sackmann (1995). In this model, cytoskeleton as well as glycocalix could decrease the lateral diffusion of lipids and therefore induce their micro-compartmentalization. This has been widely documented in the case of the cortical actin networks and is known as the membrane-skeleton “fence” (Kusumi et al., 2012).

Although all these membrane models give many possible explanation of the observed lateral heterogeneity and domains existence in lipid membrane, the most popular in bioscience nowadays is based on lipid demixing and named the “rafts” model. Initially reported by van Meer and Simons (1988), “rafts” were considered to be microdomains (*r* ~ 100–300 nm) enriched in sphingolipids and cholesterol that are functionally associated to specific proteins involved in trafficking and cell signaling (Simons and Ikonen, 1997). Since then, “rafts” spawned thousands of projects and papers up to a point where nowadays a membrane protein is often classified as being a “raft” or a “non-raft” component of the membrane. However, accurate physical explanation of the “raft” hypothesis is still lacking and its definition has been revisited many times to end up with the most recent one as being “fluctuating nano-scale assemblies (*r* ~ 20–50 nm) of sphingolipid, cholesterol and proteins that can be stabilized to coalesce, forming platforms that function in membrane signaling and trafficking” (Lingwood and Simons, 2010). They are claimed to exist in an ordered phase (or “raft-phase”) different from the liquid ordered phase observed in model membrane systems. The thermodynamic term phase relies on a system at equilibrium and it remains to be established if the plasma membrane is near local equilibrium at some time scales in order to permit real phase separation. Another problem with the “raft” hypothesis is that they have mainly been observed using detergent based extraction methods. It is clear that these methods will always isolate from biological membranes the proteins (and their associated lipids) partitioning into the detergent, thereby inducing formation of domains. Even Lingwood and Simons themselves concluded that detergent extraction methods do not isolate pre-existing membrane domains (Lingwood and Simons, 2010). Finally, while conclusive experiments about the existence of rafts in the plasma membrane remain elusive it is clearly established (Ipsen et al., 1987) that, in model systems containing cholesterol, liquid ordered (lo) and liquid disordered (ld) phase coexists. It is also important to state that, as originally proposed by Sackmann (1995), cytoskeleton (Ehrig et al., 2011a; Sens and Turner, 2011) as well as trafficking (Turner et al., 2005) could play a major role in the lateral segregation of lipids.

Biological lipid membranes are not only characterized by their lateral heterogeneity but also by their asymmetric transverse lipid distribution. Each of the plasma membrane monolayer (outer and inner) significantly differ in their chemical composition. It is generally accepted that the outer leaflet is enriched in sphingolipids (SL) and phosphatidylcholine (PC) whereas the inner leaflet is enriched in phosphatidylethanolamine (PE), phosphatidylserine (PS), and phosphatidylinositols (PI, PIP, PIP_2_, PIP_3_). This transverse asymmetry can also be defined in term of acyl chains saturation/unsaturation distribution. Indeed, mono or poly-unsaturated acyl chain are mainly found in PE, PS and PI(P)_*x*_ whereas saturated ones are esterified on PC and SL. The transverse partitioning of cholesterol is unclear but surprisingly seems to be in favor of the cytoplasmic leaflet in different cells (Devaux and Morris, 2004; Wood et al., 2011). These observations point out key questions about the existing lateral heterogeneities in the plasma membrane. How can a “raft” exist in the inner leaflet of the plasma membrane since it is depleted in sphingolipids and enriched in unsaturated acyl chains? Can lipid domains of different nature and composition exist in both leaflets? Finally, if they do exist, how are they coupled? Indeed, there is no theoretical problem with the existence of Lo domains in both leaflets of the plasma membrane, although the physical and chemical properties of these domains must be different. For example, liquid ordered (Lo) domains could exist in PS/PE dominated inner leaflet, provided these lipids are saturated or bear only one unsaturated fatty acid restricted to the *sn-2* position. Indeed several papers have reported that the inner leaflet domains containing PC/Chol or PE/Chol would be less stable than the outer leaflet “rafts.” This has also been observed in model membranes (Samsonov et al., 2001). Thus Lo domains in the outer and the inner membrane leaflet of biomembranes should not necessarily spontaneously match to each other. On the opposite, some studies performed on asymmetric model system at the thermodynamic equilibrium tend to show the opposite result and end up in a coupled macroscopic phase separation (Lo/Ld) on both leaflets (Allender and Schick, 2006; Wan et al., 2008; Kiessling et al., 2009).

### Molecular shape and curvature

**Figure 1:**
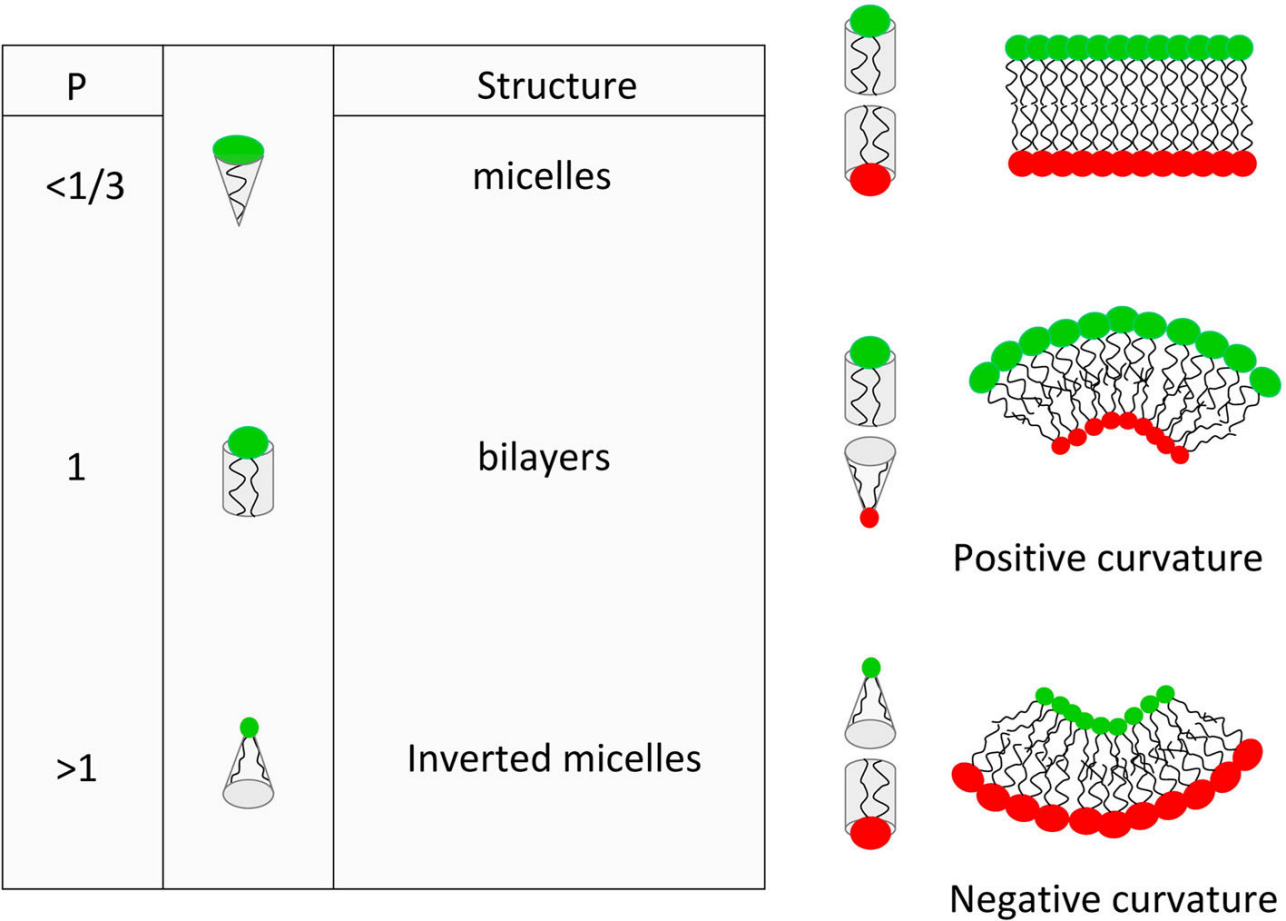
**Basic concepts of the lipid average molecular shape and its effect on spontaneous curvature of lipid membranes**.

Another topic of interest for retroviral assembly is the role of spontaneous local curvature of the membrane. A major regulator of this local curvature is the lipid average molecular shape (Israelachvili, 1977). This molecular shape can be defined by a simple geometric property of the molecule (Israelachvili-Mitchell-Ninham packing parameter: *P* = *v/al*), where *v* is the molecular volume, *a* is the cross section area of the head group and *l* is the length of the molecule (mainly due to acyl chains) (see Figure 1). In a dynamic aggregate, those values should be considered as average molecular properties. Although the role of the average molecular shape in the spontaneous curvature of lipid bilayers is more and more questioned (Cooke and Deserno, 2006), value of *P* turns out to be very useful to predict the structure of lipid assemblies. For example, it is clearly seen from Figure 1 that if one of the two leaflets of the membrane start having different average *P* value, the bilayer will suffer from built-in curvature stress.

Another way to describe the membrane curvature in a continuous model is the bending energy. This latter relies on both the spontaneous curvature (which can be seen as the average value of the molecular shape) and the bending stiffness of the membrane (basically, the thicker the membrane, the higher the bending stiffness). It is clear that average curvature plays a role in lipid sorting although it is hard to define if lipid sorting induce curvature or the opposite (for an extensive review see Callan-Jones et al., 2011). Nevertheless, many reports have shown that bending energy of a homogeneous tri-component model membrane submitted to inhomogeneous curvature can be reduced by enriching the highly curved region in liquid disordered lipids (van Meer and Lisman, 2002; van Meer and Sprong, 2004). It then appears that lipid sorting induced by curvature is a collective effect more than a single molecule effect. Moreover, it is clear that membrane attached proteins (such as Gag) plays a role in this average curvature and the associated lipid sorting. It is therefore highly probable that HIV-1 virus budding, which induces a positive curvature of the membrane, should occur with different average molecular shape on both leaflets of the membrane, i.e., with different lipid composition on both leaflet. In their review (Callan-Jones et al., 2011) stated that “In cell membranes, it is very unlikely that individual lipids, unassisted by interactions with themselves or with proteins, can be enriched in curved regions simply based on their shape alone.” This questions the role of individual molecular spontaneous curvature in the curvature based lipid sorting in cells and on the opposite, this reinforce the role of proteins and lipid domains in inducing or responding to curvatures.

To summarize, on both side of the membrane, lipid domain formation and stabilization are due to a combination of lateral segregation or phase separation, induced or spontaneous curvature, transverse distribution asymmetry. This has been recently shown in the outer leaflet in the case of “raft” domains (Meinhardt et al., 2013).

## Role of lipids domains in HIV-1 assembly

As stated in the introduction, the minimal component required for HIV-1 assembly at the plasma membrane, is the viral Gag protein. Its MA domain is mainly responsible for membrane interaction and targeting to the plasma membrane (Ono et al., 2000), although MA alone seems to exhibit a lower affinity for membrane than Gag (Zhou and Resh, 1996). MA domain has a bipartite motif of interaction with lipid membranes like many other proteins of the same class (for review see Resh, 1999). Indeed, HIV-1 Gag interacts with acidic lipids such as phosphatidyl inositols phosphates(PIP_*x*_) and phosphatidylserine (PS) by means of a polybasic region—called HBR (Highly Basic Region)—(Zhou et al., 1994; Freed et al., 1995; Ono and Freed, 1999). Amongst the (PIP_*x*_), the phosphatidylinositol 4,5 bisphosphate (PI(4,5)P_2_) is considered to be the more specific (Ono and Freed, 2004; Chukkapalli et al., 2008; Hamard-Peron et al., 2010) but phosphatidylinositol 3,4 bisphosphate (PI(3,4)P_2_) or phosphatidylinositol 3,4,5 triphosphate (PI(3,4,5)P_2_) also bind efficiently (Anraku et al., 2010). As it is the case for other polybasic proteins (Ben-Tal et al., 1997; Murray et al., 1997), one can expect that this electrostatic interaction occurs at long range distances (*d* > 1.5 nm) and can be considered as the attractive force between the lipid membrane and the Gag protein. In addition to its HBR region, Gag is also myristoylated at its N-terminus. This myristoylation is responsible for tightening the attachment of Gag to the plasma membrane by insertion into the membrane.

Elucidation of HIV MA structure bound to diC_4_PI(4,5)P_2_ or diC_8_PI(4,5)P_2_ has shown a switch of the myristoyl from an hydrophobic pocket of MA into the membrane. This switch is thought to be induced by electrostatic binding to PI(4,5)P_2_ (Saad et al., 2006, 2007; Ono, 2010a). Nevertheless, it appeared that the myristate can spontaneously be released from its hydrophobic pocket in the vicinity of lipid membranes or upon trimerization of the protein (Tang et al., 2004; Valentine et al., 2010; Charlier et al., 2014). While Valentine et al. suggested that Gag released myristate could probe the membrane by successive insertion/exclusion until finding its PI(4,5)P_2_ target, the coarse grain molecular modeling of Charlier et al. (2014) suggest in contrast, that insertion of the myristate occurs after non-specific electrostatic attraction to the membrane and permits the Gag protein to find a correct orientation to capture the PI(4,5)P_2_ head in the HBR. Recent NMR data obtained by Vlach and Saad (2013) suggested that other lipid such as PS, PE and PC could reinforce the interaction of Gag with plasma membrane by direct binding to a different site of Gag.

The role of pre-existing lipid domains in the interaction of Gag with the plasma membrane and in HIV-1 assembly has been studied for many years.

Different experiments based on:

- detergent solubilization (Nguyen and Hildreth, 2000; Lindwasser and Resh, 2001; Ono and Freed, 2001; Holm et al., 2003)
- cholesterol depletion (Ono and Freed, 2001; Ono et al., 2007)
- immunofluorescence co-localization (Nguyen and Hildreth, 2000; Holm et al., 2003; Ono et al., 2005)
- lipidomics (Bruegger et al., 2006; Chan et al., 2008; Lorizate et al., 2013).

have suggested a potential role of “rafts” in the assembly of Gag. Although lipidomic studies differ one from each other, they have mainly shown that HIV lipid envelope was highly enriched in PIP_*x*_, but also slightly in sphingomyelin (Chan et al., 2008; Lorizate et al., 2013). Results are contradictory regarding enrichment in cholesterol and PS (Chan et al., 2008; Lorizate et al., 2013).

TEM have also been proposed to be the site for HIV-1 assembly (Booth et al., 2006; Nydegger et al., 2006; Thali, 2009). TEM has been shown to co-localize with Gag in T cells (Jolly and Sattentau, 2007; Grigorov et al., 2009) and tetraspanin components are found to be incorporated into HIV-1 particles, especially CD81 (Grigorov et al., 2009). GM3, for example, is described to be highly present in TEM (Hemler, 2005; Yanez-Mo et al., 2009) and is enriched in the virus lipid envelope compared to the plasma membrane (Chan et al., 2008).

Although the functional goal of assembling into TEM or “rafts” has still not been elucidated, different molecular mechanisms (mainly for assembly into “rafts”) have been proposed. Moreover it has been suggested that Gag could induce the coalescence of clustered rafts and TEMs at its own assembly site (Hogue et al., 2011).

“Rafts” are considered to be mainly enriched in saturated lipids, therefore the major problem of Gag partitioning into “rafts” is its interaction with PI(4,5)P_2_, which naturally bear a long unsaturated acyl chain in its sn-2 position. In our opinion, the most subtle, elegant and detailed model to solve this controversy is coming from the NMR structure of MA with di-C_8_PI(4,5)P_2_ (Saad et al., 2006). In this mechanism, the unsaturated sn-2 acyl chain of the PI(4,5)P_2_ is sequestered in a hydrophobic cluster of MA amino acids concomitantly to the myristate switch, whilst the saturated sn-1 acyl chain remains in the plasma membrane. The sequestration of the unsaturated fatty acid of the PI(4,5)P_2_ out of the hydrophobic part of the membrane thereby allows the complex MA-PI(4,5)P_2_ to partition into rafts (Ono, 2010b; Simons and Gerl, 2010). This model has been recently reinforced by the suggestion that Gag could sense cholesterol and liquid-ordered acyl-chains environments (Dick et al., 2012) and by new NMR experiments performed by Vlach and Saad (2013). These new NMR data show the existence in MA of a second lipid binding site inducing sn-2 acyl chain flipping into a new associated hydrophobic pocket whatever the bound lipid is (PS, PC, PE). Since sn-2 position is usually the place where unsaturated acyl chains hold in lipids, this new model shows that MA is able to locally deplete the complex MA-bound lipids of unsaturated acyl chains. As a result, the complex, mainly bearing saturated acyl chains, could therefore partition faster into lipid “rafts” despite the lack of direct interaction with sphingomyelin (Vlach and Saad, 2013).

Nevertheless partitioning of Gag into “rafts” is still a matter of controversy since “rafts,” as they are defined, can almost exclusively exist in the outer leaflet of the plasma membrane, explaining therefore the lack of direct interaction of MA with sphingomyelin. Recently, Keller et al. (2013) have nicely shown that a myristoylated multimerizable Gag bound to PI(4,5)P_2_ containing model membrane exclusively partition into liquid disordered domains, not ordered (“rafts”) ones. Moreover, starting from the NMR structure established by Saad et al. (2006), Charlier et al. (2014) have performed coarse grain molecular dynamics of Myr-MA in the presence of a lipid bilayer whose composition approach the inner leaflet of the plasma membrane. Our study shows that, in this configuration, the unsaturated sn-2 acyl chain of the PI(4,5)P_2_ never flipped out of the membrane into an hydrophobic pocket of Gag. It is important to notice that, although it is not discussed in the paper of Charlier et al. (2014), PS was seen to bind at the site where Saad et al. have seen it by NMR (Vlach and Saad, 2013), but, here again, without any flipping of its sn-2 acyl chain. Moreover, in the light of what is described on page 3 of this review, it is worth wondering how interesting it would be to trap acyl chains into hydrophobic pockets of Gag during HIV-1 assembly. An oversimplified guess shows that removing acyl chains from the plane of the membrane will locally change the molecular curvature of the complex and will induce, during assembly, a negative curvature opposite to the positive curvature expected for budding. Whereas there is no doubt that the viral lipid envelop is enriched in sphingomyelin and cholesterol it is still unclear at which step of the assembly this enrichment occurs. Indeed, these two recent studies clearly questions the role of “raft” as a pre-existing platform where virus assembly occurs.

A question that still remains is the possibility for Gag to be targeted at pre-existing inner leaflet domains. Some studies have suggested that PI(4,5)P_2_ could spontaneously aggregate into nanodomains (Johnson et al., 2008; Ellenbroek et al., 2011; Salvemini et al., 2014). But it has also been shown that PI(4,5)P_2_ is sequestered by proteins in the cell (for review see McLaughlin et al., 2002). In our opinion, based on different other studies regarding the effect of membrane bound proteins on lipid phase separation (Ehrig et al., 2011a,b; Witkowski et al., 2012), it is more than likely that, as we already proposed (Kerviel et al., 2013), assembly induces lipid domain, not the opposite. Using coarse grained molecular dynamics of the interaction of Myr-MA with inner lipid leaflet we have shown a potential enrichment of PI(4,5)P_2_ all around the protein (Charlier et al., 2014), leading to putative enriched acidic lipid nanodomain formation as we already suggested in Kerviel et al. (2013).

Based on micro-emulsion theory, it has been recently demonstrated (Shlomovitz and Schick, 2013) that local fluctuations of curvature could induce asymmetric lipid domains (in term of lipid composition) in both leaflet. The theory predicts that inner PS enriched domains could face outer SL enriched domains (“rafts”), which turns out to be very nice in terms of HIV-1 assembly… Unfortunately, in this configuration (PS domains facing SL domains), the induced curvature is negative, i.e., opposite to viral budding. More generally, the role of pre-existing lipid domains in favoring virus assembly is unclear, whatever their composition and origin are. Indeed, a recent study on the dynamics of the interaction of Gag with TEM domains has shown Gag multimerization to be responsible for trapping CD9 into the domain of assembly instead of Gag targeting through CD9 on preexisting TEMs (Krementsov et al., 2010). This suggests that during retroviral assembly, Gag is trapping membrane components instead of being trapped at specific pre-existing domains. These controversial data shows that the role of lipids during HIV-1 assembly is far from being elucidated.

## Conclusion

One of the key questions regarding the role of membranes in HIV-1 assembly is the time-ordering of events across the membrane. Is there an induction of inner leaflet lipid domains during multimerization process domains or is Gag targeted to pre-existing coupled outer and inner leaflet domains in order to rapidly assemble. Indeed, in the released virus, the ratio Gag to Env is largely in favor of Gag, it seems therefore reasonable to expect that HIV-1 assembly is an “inside out” process, not an “outside in,” i.e., Gag may be driving the assembly from the inside, not pre-existing outer “rafts” domains with Env trapped into (for a scheme of the process see Figure 1 in Mariani et al. in this special issue). Nevertheless, at the moment, it appears that the respective roles of lipid domains and viral proteins during HIV-1 assembly are still entangled. It thus remains an exciting challenge for virologists as well as for biophysicists to remove this degeneracy.

### Conflict of interest statement

The authors declare that the research was conducted in the absence of any commercial or financial relationships that could be construed as a potential conflict of interest.
